# Structural Determinants of the 5′-Methylthioinosine Specificity of *Plasmodium* Purine Nucleoside Phosphorylase

**DOI:** 10.1371/journal.pone.0084384

**Published:** 2014-01-08

**Authors:** Teraya M. Donaldson, Li-Min Ting, Chenyang Zhan, Wuxian Shi, Renjian Zheng, Steven C. Almo, Kami Kim

**Affiliations:** 1 Departments of Medicine, Pathology, and of Microbiology & Immunology, Albert Einstein College of Medicine, Bronx, New York, United States of America; 2 Department of Biochemistry, Albert Einstein College of Medicine, Bronx, New York, United States of America; 3 National Synchrotron Light Source, Brookhaven National Laboratory, Brookhaven, New York, United States of America; 4 Department of Molecular Pharmacology, Albert Einstein College of Medicine, Bronx, New York, United States of America; Johns Hopkins Bloomberg School of Public Health, United States of America

## Abstract

*Plasmodium* parasites rely upon purine salvage for survival. *Plasmodium* purine nucleoside phosphorylase is part of the streamlined *Plasmodium* purine salvage pathway that leads to the phosphorylysis of both purines and 5′-methylthiopurines, byproducts of polyamine synthesis. We have explored structural features in *Plasmodium falciparum* purine nucleoside phosphorylase (PfPNP) that affect efficiency of catalysis as well as those that make it suitable for dual specificity. We used site directed mutagenesis to identify residues critical for PfPNP catalytic activity as well as critical residues within a hydrophobic pocket required for accommodation of the 5′-methylthio group. Kinetic analysis data shows that several mutants had disrupted binding of the 5′-methylthio group while retaining activity for inosine. A triple PfPNP mutant that mimics *Toxoplasma gondii* PNP had significant loss of 5′-methylthio activity with retention of inosine activity. Crystallographic investigation of the triple mutant PfPNP with Tyr160Phe, Val66Ile, andVal73Ile in complex with the transition state inhibitor immucillin H reveals fewer hydrogen bond interactions for the inhibitor in the hydrophobic pocket.

## Introduction

**Figure 1:**
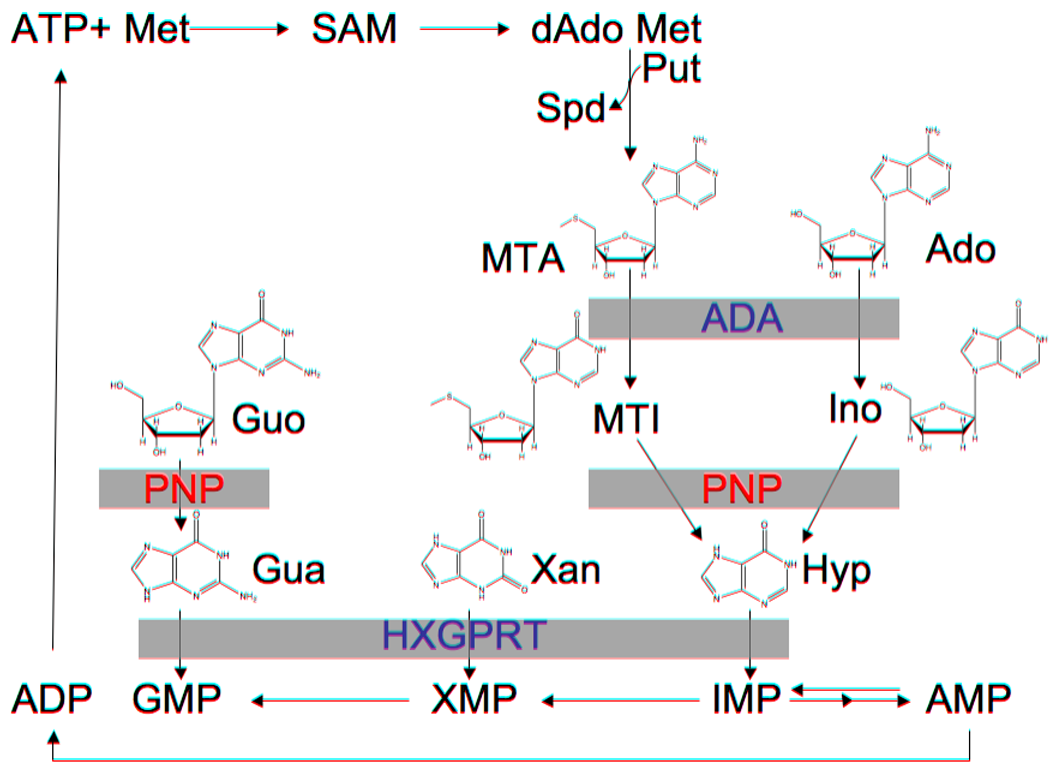
Purine salvage in *P. falciparum*. The enzymes that comprise the purine salvage pathway in *Plasmodium*: ADA, adenosine deaminase; PNP, purine nucleoside phosphorylase; HXGPRT, hypoxanthine-xanthine-guanine phosphoribosyltransferase. Substrates are: MTA, 5′methylthioadenosine; Ado, adenosine; MTI, 5′methylthioinosine; Ino, inosine; Hyp, hypoxanthine; IMP, inosine monophosphate; AMP, adenosine monophosphate; XMP, xanthine monophosphate; GMP, guanosine monophosphate; ATP, adenosine triphosphate; SAM, *S*-adenosylmethionine; SAH, S-adenosylhomocysteine.

Malaria, caused by *Plasmodium spp*, continues to be an important public health problem for which new interventions are needed. While much progress has been made in malaria control, in 2010 there were an estimated 219 million clinical cases estimated worldwide, with 660,000 deaths primarily in children in sub-Sarahan Africa [1]. Because *Plasmodium* is unable to synthesize purines *de novo*, *Plasmodium* purine salvage enzymes have been investigated as potential chemotherapeutic targets. Unlike many other protozoa, *Plasmodia* have a streamlined purine salvage system consisting of adenosine deaminase (ADA)+purine nucleoside phosphorylase (PNP)+hypoxanthine-xanthine-guanine phosphoribosyltransferase (HXGPRT) (Figure 1) [2]. PNP catalyzes the phosphorylytic cleavage of purine nucleosides to ribose-1-phosphate and a purine base [3]. PfADA converts adenosine to inosine. PfPNP converts inosine or guanosine to hypoxanthine or guanine that is then acted upon by HXGPRT to generate IMP or GMP. Hypoxanthine is the major purine precursor utilized by *Plasmodium*.

Polyamine synthesis pathways are also critical for *Plasmodium* viability [4]–[8] and generate 5′-methylthioadenosine (MTA) as a byproduct of polyamine synthesis. Humans recycle purines from MTA via the action of methylthioadenosine phosphorylase (MTAP) but *Plasmodium* species recycle purines via the sequential activities of ADA and PNP, which are unique in their ability to utilize methylthiopurines [9]. In *P. falciparum*, PfADA converts MTA to 5′-methylthioinosine (MTI), which is then converted to hypoxanthine by PfPNP. Humans do not produce MTI, and human PNP does not catalyze the phosphorolysis reaction of methylthioinosine [10].

**Figure 2:**
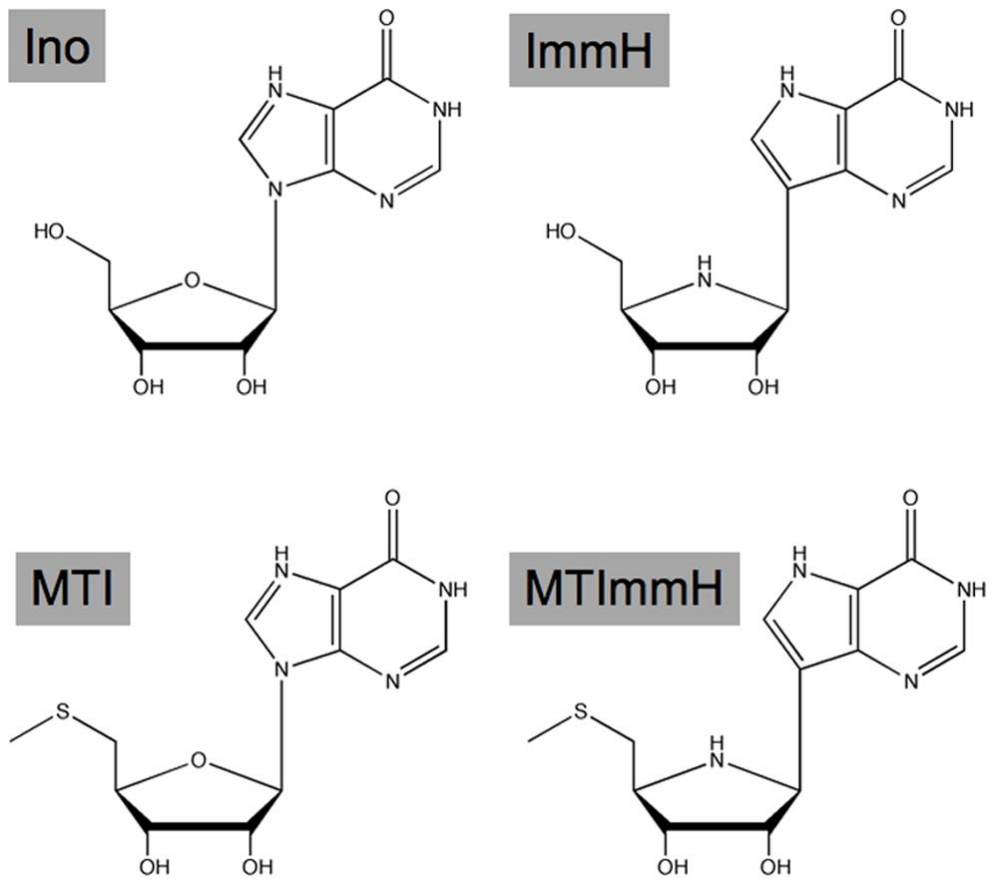
PfPNP substrates and inhibitors. Structures of substrates (inosine and 5′-methylthioinosine) and immucillin transition state analogues (ImmH and MT-ImmH) of PfPNP utilized for this study.

The unique dual specificity of *P. falciparum* PNP can be exploited for anti-malarial drug design. Immucillin-H (ImmH) and 5′-methylthioimmucillin-H (MT-ImmH) are transition state analogs of inosine and MTI, respectively (Figure 2). Immucillins are extremely potent with picomolar *K_d_* for PNPs [4], [5], [11], [12]. In the purine-rich environment of cultured red blood cells, ImmH causes *P. falciparum* cell death by purine starvation [2]. MT-ImmH exhibits 100-fold greater specificity for PfPNP versus mammalian PNP [13]. Genetic studies have revealed that *Plasmodium* parasites lacking PNP are attenuated [14], [15], demonstrating the importance of this enzyme for viability of malaria parasites. The genetic studies also validated PNP as the target of immucillins [14], [15]. In addition, DADMe-Immucillin-G a picomolar transition state analogue of human and *Plasmodium* PNPs is effective against *P. falciparum* in the *Aotus* model, illustrating that purine salvage is critical for *P. falciparum* survival [16].

PfPNP, like *Escherichia coli* PNP, is hexameric and a member of the nucleoside phosphorylase family I [3]. Unexpectedly, the PfPNP crystal structure revealed that the 5′-hydroxyl group of ImmH and 5′-methylthio group on the MT-ImmH are positioned differently in relationship to PfPNP [17]. The 5′-methylthio of MT-ImmH is rotated ∼135° when compared to the 5′-hydroxyl group of ImmH, and therefore the residues that surround the 5′-group are different [17]. If *Plasmodium*-specific PNP inhibitors are developed further, the capacity of the parasite to develop resistance to new agents must be explored. Thus the structural features of PNP responsible for inosine and MTI metabolism are of great interest.

The purine and polyamine pathways of the related apicomplexan *Toxoplasma gondii* have significant biologically relevant differences to those of *Plasmodium* [18], [19]. While *Plasmodium* species must synthesize polyamines, *T. gondii* salvages polyamines from host cells and therefore does not require enzymes to metabolize MTA [18]. Consistent with this, TgPNP does not catalyze MTI conversion to hypoxanthine [18].

**Figure 3:**
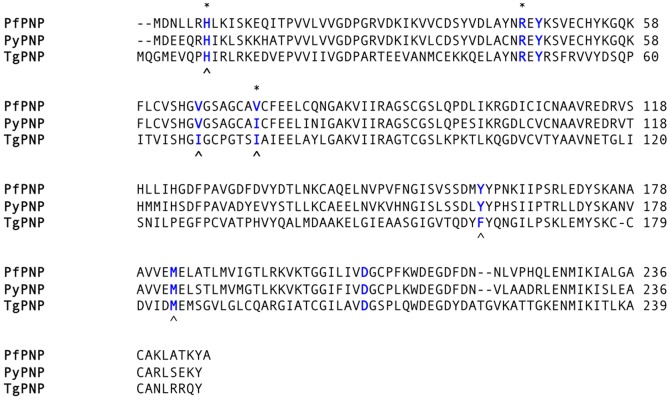
Alignment of apicomplexan PNPs. ClustalW alignment of PNP protein sequences from *T. gondii* (TgPNP), *P.yoelli* (PyPNP), and *P. falciparium* PNP (PfPNP). Residues involved in substrate binding are highlighted [38]. Residues in blue font indicate those surrounding the catalytic domain that were mutated in this study. Amino acids marked: (*) are from the adjacent subunit, (∧) residues are associated with the hydrophobic cavity for accepting the 5′-Methylthio group of MTI.

We hypothesized that the differences between TgPNP and PfPNP would enable us to determine the unique structural features responsible for 5′-methylthiopurine activity. After comparison of the amino acid sequences of apicomplexan PNPs (Figure 3) with the PfPNP crystal structure [17], we identified conserved and nonconserved residues potentially critical for catalytic activity. We made a series of PfPNP mutants and performed detailed kinetics and structural studies. In particular, PfPNP mutants with activity for inosine but not MTI provided clues as to the malleability and conformation of the active site, providing insights that may be useful for future design of anti-malarial compounds.

## Materials and Methods

### Reagents

Xanthine oxidase, inosine, ampicillin, IPTG and protease inhibitor cocktail were purchased from Sigma (St. Louis, Mo). Ni-NTA agarose was purchased from Qiagen (Valencia, CA). 5′-methythioinosine was generated from MTA using PfADA as described [13]. ImmH and MT-ImmH were synthesized as described previously [6], [17] and were the generous gift of Peter Tyler, Gary B. Evans and Vern Schramm. Crystallography reagents and plates purchased from Hampton Research (HR2-110 and HR3-297) (Aliso Viejo, CA).

### Site Directed Mutagenesis

The gene for PfPNP of *P. falciparum* (AF426159; PFE0660c) is located on chromosome 5. Its activity as PNP has been fully characterized [2], [10], [17], [20]. PfPNP coding sequence from genomic DNA of *P. falciparum* 3D7 strain was subcloned into the pTrcHis2-topo expression plasmid [13] and used as a template for mutagenesis. PfPNP mutants were generated by polymerase chain reaction (PCR) using the QuikChange Multisite-directed mutagenesis kit from Stratagene (La Jolla, CA). Mutants were selected based on their location in the active site from wild-type PfPNP (PDB code 1NW4 and 1Q1G). The oligonucleotides used for mutagenesis are listed in Table S1. All point mutants were analyzed and confirmed via automated DNA sequencing (Albert Einstein College of Medicine DNA sequencing facility, Bronx, USA). Combinations of the Val66Ile, Val73Ile, and Tyr160Phe primers were used to make double (Val66Ile,Val73Ile; Val66Ile:Tyr160Phe, Val73Ile:Tyr160Phe) and triple mutations (Val66Ile:Val73Ile:Tyr160Phe).

### Cloning and expression of P. falciparum PNP mutants

PfPNP and mutants were expressed in *Escherichia coli* Top 10 cells as previously described in [13]. One hundred milliliter cultures were grown in LB-ampicillin media at 37°C until A_595_ = 0.6 and then induced with 1 mM isopropyl 1-thio-ß-D-galactopyranoside (IPTG) for 6 hrs or overnight. The cell cultures were sonicated in 10 mM imidazole lysis buffer (50 mM NaPO_4_, 1 mM ß-mercaptoethanol, 300 mM NaCl, at pH 8) with protease inhibitor cocktail (Sigma) and 1 mg/ml lysozyme while on ice. After centrifugation at 12000 rpm for 30 minutes, cleared cell lysate was shaken at 4fiC with Ni-NTA resin for 1 hr, and then packed into a column. The column was washed twice with 50 mM imidazole buffer (50 mM NaPO_4_, 300 mM NaCl, at pH 8), and eluted with 250 mM imidazole buffer (50 mM NaPO_4_, 300 mM NaCl, at pH 8). The purity of the proteins (>95%) was confirmed by SDS page gel electrophoresis (data not shown). Protein concentration was measured using protein assay reagent from BioRad (Hercules, CA).

### Enzymatic Assays and Inhibition Studies

Kinetic assays were completed in 50 mM KH_2_PO_4_ at pH 7.4 measuring phosphorylysis of inosine or MTI by PNP in a coupled reaction with 60 milliunits/ml xanthine oxidase to convert hypoxanthine to uric acid. Formation of uric acid was measured at 293 nm wavelength (E_293_ = 12.9 mM^−1^ cm^−1^) [2], [20]. In cases where activity for MTI was low, increased protein concentration (10 µg/ml) was used to check enzyme function. Assays were performed with excess substrate in the presence of inhibitors. Inhibition studies measured both initial (*K_i_*) and slow onset inhibition (K_i_*) for inhibitors [2]. The initial onset inhibition was analyzed by using the following equation: *ν*_ο_ = (*k*_cat_×S)/(*K*_m_(1+*I*/*K_i_*)+S), *ν*_ο_ is the steady state rate, *k*_cat_ catalytic rate, S is substrate concentration, *K_m_* is Michealis constant for inosine, I is inhibitor concentration, and *K_i_* is the equilibrium dissociation constant.

### Molecular Modeling

Model of site-directed mutants were comparatively designed based on the X-ray crystallography structure of *P. falciparum* purine nucleoside phosphorylase PfPNP [17] bound to MT-ImmH (Protein Data Bank entry 1Q1G). Point mutant structures were created utilizing MODELLER 8v2 program [21], [22]. Structural visualization was performed using the PyMOL molecular graphics program [23].

### Gel Filtration

Samples were concentrated to 1 mg/ml and 0.1 ml was loaded onto a Superose 12 gel filtration column for initial screening. The enzyme was eluted with 20 mM HEPES containing 50 mM KCl, 5 mM KH_2_PO_4_, 0.1 mM DTT (pH 7.4) a rate of 0.5 ml/min. Larger scale purification was performed on a Superdex 200 gel filtration column with a sample concentration of 10 mg/ml with 5 ml loaded. Both columns were run on an AKTA FPLC (GE Healthcare).

### Circular Dichroism Analysis

Secondary structure measurements were taken from 300–185 nm on Aviv 215 CD spectrometer at Mount Holyoke College in the laboratory of Dr. Sean Decatur (Figure S1). The experiments were performed at 4°C in a Peltier temperature-controlled cell chamber. Samples were equilibrated for 10 minutes before measurements were taken. The averaging time for each wavelength was 10 seconds. Enzyme concentration for each run was 0.1 mg/ml (3.3 nM). Spectra were converted to molar ellipticity (θ) after subtracting the baseline values of 10% HEPES buffer measured at 4°C. Molar ellipticity conversion = millidegrees/(pathlength×number of residues×molar protein concentration).

### Crystallization and X-ray methods

Diffraction quality crystals of PfPNP V66I:V73I:Y160F•ImmH were grown by sitting drop vapor diffusion at 18°C by mixing 2 µl of 10 mg/ml *P. falciparum* PNP with ImmH (1∶1.5 molar equivalents) and 1 µl of reservoir solution consisting of 0.2 M magnesium chloride hexahydrate, 0.1M HEPES at pH 7.5, and 30% 2-propanol. The drop was equilibrated with 100 µl of reservoir solution. Crystals with a cubic habit appeared after one month. The crystals were cryo-protected in mother liquor supplemented with 15% glycerol and flash cooled in liquid nitrogen. Data were collected at Brookhaven National Lab National Synchrotron Light Source at beam line X29A with an ADSC Quantum 315 detector. Diffraction data were collected to a resolution of 2.8 Å, and integrated and scaled with HKL2000 [24]. Diffraction from the PfPNP V66I:V73I:Y160F •ImmH•PO_4_^3−^ crystals was consistent with the cubic space group I4_1_32, with unit cell parameters a = b = c = 234.97 Å, α = β = γ = 90° and 2 molecules in the asymmetric unit. An initial structure of PfPNP V66I:V73I:Y160F •ImmH•PO_4_^3−^ complex was determined by molecular replacement with the program PHASER [25], using the PfPNP•ImmH•SO_4_^2−^ structure (1NW4) as the search model. The final model was refined with REFMAC5 [26]. PfPNP V66I:V73I:Y160F •ImmH•PO_4_^3−^ complex PDB ID is 3FOW.

## Results

Based on the conservation of residues in the sequence alignment of PNPs and the PfPNP crystal structure, we identified several residues that were predicted to be critical for catalytic activity. PfPNP and PyPNP activities are enzymatically indistinguishable with conservation of most catalytic residues [14], but, notably, there are residues within the hydrophobic pocket in contact with the 5′-methylthio group that are different in TgPNP (Figure 3). Of the five residues that surround the 5′-methylthio group in PfPNP, there are three substitutions in TgPNP. His7 and Met183 are conserved in human PNP, *E. coli* PNP as well as *T. gondii* and *Plasmodium* PNPs. The PfPNP residues Val66, Val73, and Tyr160 correspond to residues in TgPNP Ile68, Ile75, and Phe162. We tested whether these three residues are determinants in the accommodation of the 5′-methylthio group in the active site of PfPNP [17].

### Characterization and Purification of PfPNP

Using site-directed mutagenesis, recombinant PfPNP mutants were created, over-expressed and nickel affinity-purified from *E. coli*. Mutant and wild-type preparations were analyzed by SDS PAGE gel, and final protein purity was estimated to be >95% (data not shown). Both wild-type and mutant PfPNPs have a 6xHis and C-Myc tag at the C-terminus [20]. Mutants show similar migration on SDS-PAGE and prior studies showed that neither 6xHis nor C-Myc tag interfere with activity but enable rapid efficient purification [10], [13], [17].

### Kinetic Activity of Wild-type PfPNP and Active Site Mutants

Conserved residues in contact with the 5′ group of the substrate and residues critical for catalysis were mutated to alanine (Table 1). His7 and Met183 are conserved residues involved in binding substrate, whereas Arg45 is a conserved residue involved in phosphate binding [27]. Asp206 is proposed to be the general acid/base for protonation of N7 of substrate in the transition state.

**Table 1:** Kinetic constants for mutant and wild type PNPs from *P. falciparum* and *T. gondii*.

	Inosine			5′-Methylthioinosine			Ino/MTI
PNP	*K_m_*	*k_cat_*	*k_cat_/K_m_*	*K_m_*	*k_cat_*	*k_cat_/K_m_*	*k_cat_/K_m_* Ratio
	µM	s^−1^	M^−1^ s^−1^	µM	s^−1^	M^−1^ s^−1^	
**PfPNP**	11±5	1.7±0.7	1.5×10^5^	8.8±0.2	0.83±0.03	9.4×10^4^	1.6
**His7Ala** a **^,^** b **^,^** c	45±7	1.1±0.5	2.5×10^4^	2.6±2.0	0.29±0.21	1.1×10^4^	2.2
**His7Ser** a **^,^** b **^,^** c	14±3	0.5±0.3	3.8×10^4^	3.2±2.7	0.88±0.30	2.7×10^5^	0.1
**His7Phe** a **^,^** b **^,^** c	353±22	2.2±0.7	6.5×10^3^	9.6±8.0	0.21±0.03	2.2×10^4^	0.3
**Arg45Ala** a **^,^** b	470±291	0.05±0.03	1.1×10^2^	(-)	(-)	(-)	
**Tyr47Ala**	200±0	0.04±0.00	1.9×10^2^	960±0	0.02±0	2.1×10^1^	9.3
**Val66Ala** c	13±10	2.4±0.6	1.8×10^5^	0.3±0.4	0.12±0.04	3.5×10^5^	0.5
**Val66Ser** c	78±4	1.4±1.7	1.8×10^4^	0.7±0.7	0.21±0.05	2.8×10^5^	0.06
**Val66Phe** c	1500±410	5.7±3.4	3.8×10^3^	(-)	(-)	(-)	
**Val73Ala** b **^,^** c	7±4	1.1±0.1	1.6×10^5^	20±10	1.9±0.3	9.7×10^4^	1.6
**Val73Ser** b **^,^** c	5±0	0.2±0.0	4.6×10^4^	2100±0	0.02±0	1.1×10^1^	4100
**Val73Phe** b **^,^** c	(-)	(-)	(-)	(-)	(-)	(-)	
**Tyr160Ala** c	5400±120	0.1±0.0	2.2×10^2^	200±59	0.09±0.01	4.5×10^2^	0.5
**Met183Ala** c	260±64	0.4±0.3	1.5×10^3^	(-)	(-)	(-)	
**Asp206Ala**	63±6	0.04±0.01	6.3×10^2^	(-)	(-)	(-)	

The mean (±) SD is calculated under the reaction conditions as described in the Materials and Methods section.

(-) Activity not detected under the experimental conditions.

^a^ Residues that surround the 5′-hydroxyl group of Immucillin H [17].

^b^ Residues from active site that are from adjacent subunit [17], [20].

^c^ Residues that surround the 5′-methylthio group of MT-Immucillin H [17].

The catalytic efficiency of wild-type PfPNP with inosine and 5′-methylthioinosine substrates was similar to previous reports [17] with *k*_cat_/*K_m_* values of 1.5×10^5^ M^−1^ s^−1^ and 9.4×10^4^ M^−1^ s^−1^, respectively (Table 1). Arg45Ala and Tyr47Ala PfPNP mutants have low activity for both substrates. Met183Ala has reduced activity for inosine (at 1.5×10^3^ M^−1^ s^−1^) but no detectable activity for MTI, whereas Tyr160Ala has ∼1000 fold reduced catalytic efficiency with both inosine and MTI when compared to wild-type PfPNP. Asp206Ala has low activity with both inosine and MTI.

### Substrate Specificity of PfPNP Mutants Surrounding 5′-Methylthio Group

Since the 5′- hydroxyl and 5′-methylthio groups have different orientations when bound to PfPNP, we further tested how the inosine and 5′-methylthioinosine activities could be separated. We used the amino acid sequence of TgPNP, which has negligible 5′-methylthioinosine activity [18], as a guide (Figure 3 and Table 2). Val66 and Val73 line the 5′-methylthio pocket, but Val66Ile and Val73Ile mutants and the Val66Ile:Val73Ile double mutant, show no significant change in *K_m_* or catalytic efficiency for MTI.

**Table 2:** Kinetic constants for *P. falciparum* PNP mutants simulating *T. gondii* PNP.

	Inosine			5′-Methylthioinosine			Ino/MTI
PNP	*K_m_*	*k_cat_*	*k_cat_/K_m_*	*K_m_*	*k_cat_*	*k_cat_/K_m_*	*k_cat_/K_m_* Ratio
	µM	s^−1^	M^−1^ s^−1^	µM	s^−1^	M^−1^ s^−1^	
PfPNP	11±6	1.7±0.7	1.5×10^5^	8.8±0.2	0.83±0.03	9.5×10^4^	1.6
TgPNP^a^	13±1	2.6±0.0	2.0×10^5^	31±3	0.03±0.00	9.4×10^2^	230
Val66Ile	16±4	2.0±0.2	1.3×10^5^	12 5	1.2±0.7	9.4×10^4^	1.4
Val73Ile	13±2	1.9±0.7	1.4×10^5^	11±8	0.81±0.03	7.1×10^4^	2.0
Tyr160Phe	2.5±1.2	0.21±0.02	8.3×10^4^	44±5	0.30±0.04	6.8×10^3^	12
Val66Ile/Val73Ile	28±11	4.6±0.6	1.7×10^5^	15±5	0.91±0.20	6.2×10^4^	2.7
Val66Ile/Tyr160Phe	6.2±3.5	1.2±0.16	1.9×10^5^	11±2	0.05±0.00	4.7×10^3^	39
Val73Ile/Tyr160Phe	3.6±1.3	0.59±0.13	1.6×10^5^	60±33	0.28±0.04	4.6×10^3^	35
V66I/V73I/Y160F	4.0±1.8	0.36±0.23	9.0×10^4^	18±6	0.01±0.00	5.6×10^2^	160

^a^ Values reported in Chaudhary, *et al.* [18].

The mean (±) SD is calculated under the reaction conditions as described in the Materials and Methods section.

(-) No activity detected under the experimental conditions.

PfPNP Val66Ile and Val73Ile individual and combined mutations have no discernable effect upon PfPNP catalytic efficiency for inosine (Table 2). In contrast, the Tyr160Phe mutation alone and in combination with Val66Ile or Val73Ile renders PfPNP at least 10-fold less efficient with MTI as a substrate when compared with wild-type PfPNP. Tyr160Phe PfPNP *k*_cat_/*K_m_* with MTI is 7.1×10^3^ M^−1^ s^−1^, whereas Val66Ile:Tyr160Phe and Val73Ile:Tyr160Phe *k*_cat_/*K_m_* values are 4.7×10^3^ M^−1^ s^−1^ and 4.6×10^3^ M^−1^ s^−1^, respectively. Inosine remains an effective substrate for these of mutant PfPNPs with efficiency coefficients comparable to wild-type PfPNP.

V66I:V73I:Y160F PfPNP has a 83-fold decrease in *k*_cat_ for MTI with 2-fold increase in *K_m_* (Table 2). MTI is a poor substrate for the V66I:V73I:Y160F PfPNP with catalytic efficiency 160-fold lower than for inosine (9.0×10^4^ M^−1^ s^−1^ compared to 5.6×10^2^ M^−1^ s^−1^). These kinetics are similar to those observed for TgPNP, which has <0.5% efficiency for MTI when compared to inosine [18]. Thus while Tyr160 appears to contribute significantly to the methylthio specificity of PfPNP, Val66Ile and Val73Ile mutations contribute to decrease PfPNP activity with MTI substrate.

Mutations were also made to more drastically alter the chemical properties of the residues that interact with the 5′-methylthio group (Table 1). His7, Val66 and Val73 were mutated to hydrophilic (serine) or bulky aromatic (phenylalanine) residues. These mutations resulted in greater *K_m_* and/or lower *k_cat_* with lower overall catalytic efficiency. The Val73Ser mutation had a pronounced effect on MTI activity but not inosine activity with *K_m_* of 5 µM for inosine and a *K_m_* of 2100 µM for MTI. The catalytic efficiency was 4100 fold greater for inosine (*k*_cat_/*K_m_* with inosine is 4.6×10^4^ M^−1^ s^−1^ versus MTI 1.1×10^1^ M^−1^ s^−1^), suggesting that the OH side chain of Ser perturbs the pocket critical for MT side chain binding. The bulky Val66Phe mutation in PfPNP results in *K_m_* of 1500 µM for inosine, ∼100 fold greater than for wildtype PfPNP, and this mutant has no detectable activity with MTI. Val73Phe has no activity for either inosine or MTI.

### Inhibition of PfPNP mutants by Immucillins

Immucillin H (ImmH) and 5′-methylthio-immucillin H (MT-ImmH) are potent transition state inhibitors of PfPNPs [13], [17], [18]. Immucillins bind wild-type PfPNP with 2 distinct binding stages– an initial binding, followed by slow onset tighter binding [20]. MT-ImmH was synthesized based on the transition structure of MTI [13], and inhibits PfPNP with slow onset kinetics and K_i_^*^ = 1.8 nM. MT-ImmH is more than 160-fold less effective for TgPNP with K_i_^*^ = 290 nM.

ImmH and MT-ImmH were tested against PfPNP mutants. Neither inhibitor exhibits slow onset inhibition of activity of the PfPNP mutants. In general, the inhibition constants correlate with the ability of mutants to utilize inosine or MTI. ImmH binds V66I:V73I:Y160F PfPNP more than 600 times better than MT-ImmH with *K_i_* 3.6 and 2200 nM respectively. As seen in the kinetic studies with inosine and MTI, the Tyr160Phe mutation has the greatest effect upon MT-ImmH binding. Surprisingly, Val73Ile, and Val66Ile:Val73Ile mutants bind both ImmH and MT-ImmH significantly less efficiently than wild-type PNP although enzyme kinetics with inosine and MTI remain comparable to that of wild-type. (Table 2 and 3). Val66Ile shows decreased binding for MT-ImmH, but has similar binding to WT PfPNP for ImmH.

**Table 3:** Inhibition constants for Immucillins.

	ImmH		MT-ImmH	
PNP	*K_i_* a	K_i_*	*K_i_*	K_i_*
	nM	nM	nM	nM
PfPNP WT	15	0.69	10	1.8
TgPNPWT^b^	450	2.0	7100	290
Pf Val66Ile	15	(-)^c^	31	(-)
Pf Val73Ile	280	(-)	31	(-)
Pf Tyr160Phe	0.93	(-)	1200	(-)
Pf V66I:V73I	270	(-)	180	(-)
Pf V66I:V73I:Y160F	3.6	(-)	2200	(-)

^a^ Inhibition constants for Immucillins. Assays were performed with excess substrate in the presence of inhibitors. Inhibition studies measured both initial and slow onset to ascertain the initial dissociation (*K_i_*) and to determine if a steady state exists (K_i_*) [2].

^b^ Values reported in Chaudhary, *et al.* [18].

^c^ (-) No slow onset phase was detected.

### Structure Comparisons of Wild-type and V66I:V73I:Y160F PfPNP

Wild-type PfPNP has a hexameric quaternary configuration. Since any mutation has the potential to disrupt the structural conformation of the enzyme, the mutants were assessed by gel filtration and circular dichroism. Most mutants showed similar hexameric assembly when compared to wild-type.

Circular dichroism data shows 2 local minima @ 209 nm and 223.5 nm, which reflect the alpha helical abundance determined from the x-ray crystallography data (Figure S1) [17]. Circular dichroism spectroscopy shows superimposed spectra for wild-type PfPNP, Asp206Ala, Arg45Ala, and Met183Ala single mutants. Met183Ala has a different far UV spectrum, while retaining similar spectral shape to wild-type PfPNP in the near UV region. This shift at 208 nm and the decrease of molar ellipticity intensity indicates that there is some change to the secondary nature of the enzyme when compared to wild-type PfPNP. Met183 is part of the hydrophobic pocket that forms around the 5′-methylthio group of MTI and is conserved in both the hexameric family 1 and trimeric family 2 of PNPs [3], [17]. Native protein gel, run to confirm gel filtration data, showed that Met183 has primarily a monomeric population (data not shown).

Although wild-type PfPNP and V66I:V73I:Y160F PfPNP elute similarly from the gel filtration column, V66I:V73I:Y160F PfPNP and the other mutants may have subtle conformational changes. Generally, wild-type PfPNP is stable for months at 4°C, yet V66I:V73I:Y160F PfPNP and Tyr160Phe mutants lose activity after 2-week storage at 4°C.

### Crystal Structure of V66I:V73I:Y160F PfPNP•ImmH•PO_4_^3−^

To further characterize V66I:V73I:Y160F PfPNP, we determined the structure of V66I:V73I:Y160F PfPNP bound to ImmH. WT PfPNP•ImmH•SO_4_^2−^ crystals exhibited diffraction consistent with the orthorhombic space group P2_1_2_1_2_1_ [17], whereas V66I:V73I:Y160F PfPNP•ImmH•PO_4_^3−^ (3FOW) diffraction was consistent with space group *I*4_1_32 (Table S2). Like wild-type PfPNP, the active site of V66I:V73I:Y160F PfPNP•ImmH•PO_4_^3−^ is located in the interface between two monomers. The organization of the subunits is a trimer of dimers [17], [28], [29]. The overall structures of the mutant and wild-type are similar with an RMSD of 0.3 Å for the alpha carbons calculated using Dalilite [30] comparing 1NW4 and V66I:V73I:Y160F PfPNP•ImmH•PO_4_^3−^.

**Figure 4:**
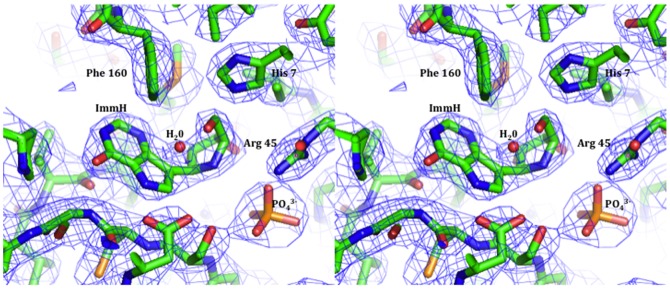
Catalytic Site of the V66I:V73I:Y160F PfPNP mutant. Cross-eyed stereo view of catalytic site of the triple mutant (V66I:V73I:Y160F) PfPNP showing bound ligand, ImmH, in a 2Fo-Fc map (blue) contoured at 1.0ó. The resolution for this map is 2.8 Å. The figure was prepared with MacPyMol [23].

The electron density map clearly supported the presence of bound inhibitor and phosphate. Figure 4 shows the ligand, ImmH, in a 2Fo-Fc map contoured at 1.0σ. Figure S2 features the omitted Fo-Fc at 3.0σ The omit Fo-Fc map was generated prior to building in the ligand. Similar to wild-type PfPNP, V66I:V73I:Y160F PfPNP active site residues are mainly contained in one monomer with His7 and Arg45 from the adjacent subunit binding 5′-hydroxyl of ImmH and the phosphate, respectively [17].

**Figure 5:**
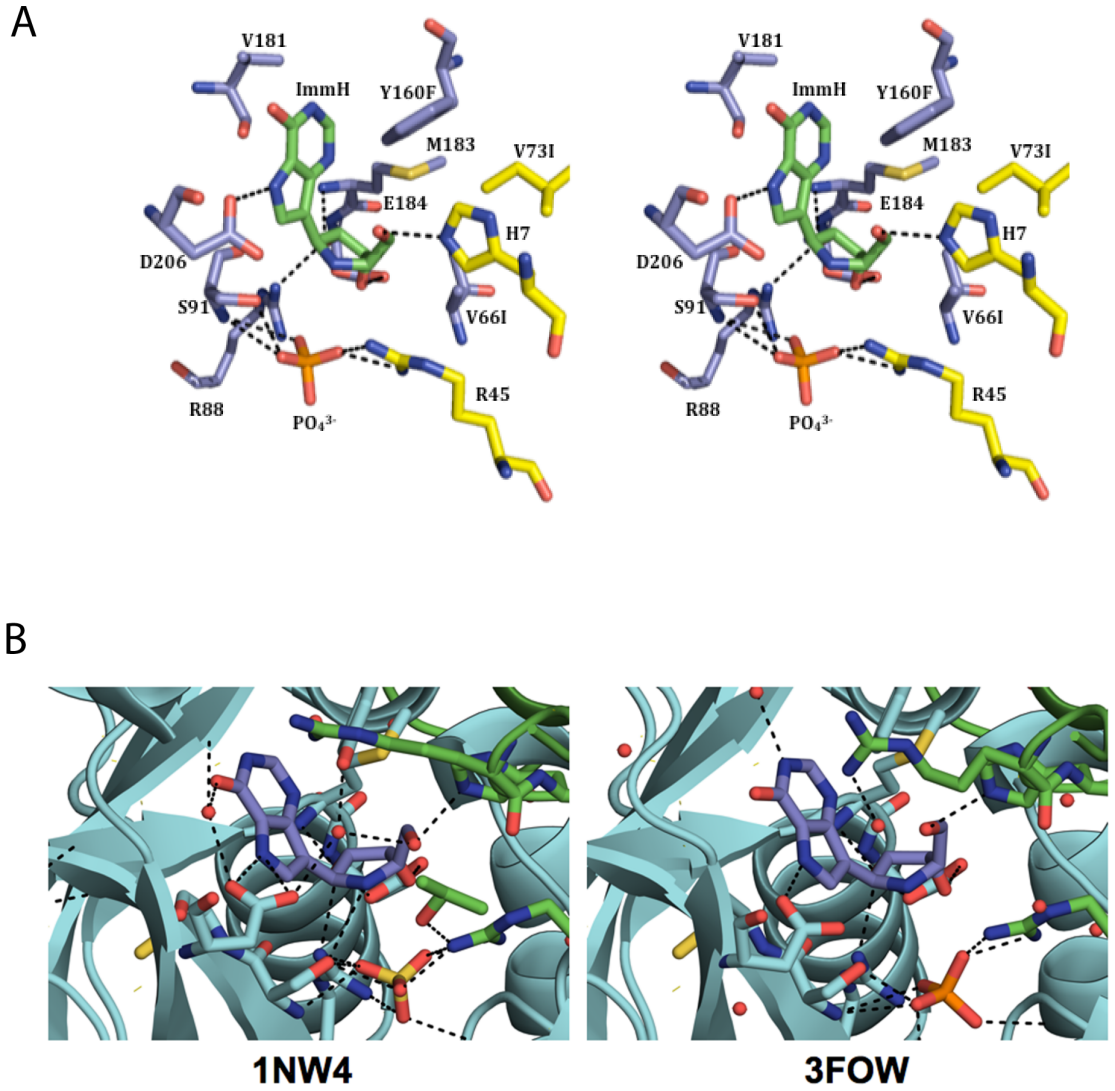
Structure of the V66I:V73I:Y160F PfPNP mutant with transition state inhibitor ImmH. **A**) Cross-eyed stereo views of the catalytic site contacts in V66I:V73I:Y160F PfPNP with the transition state analogue inhibitor ImmH and PO_4_^3−^. The figure was created using MacPyMol [23]. *Light blue* side chains show the parental monomer surrounding the bound ImmH (*green*), while the *yellow* side chains indicate residues contributed from the adjacent subunit. The highlighted imino nitrogen is in *blue* and the 5′-hydroxyl oxygen is in *red*. **B**) Side by side images of the decreased convalent interactions located in the enzymatic pocket of V66I:V73I:Y160F PfPNP mutant (3FOW) on the right with WT PfPNP (1NW4) as a comparison.

**Figure 6:**
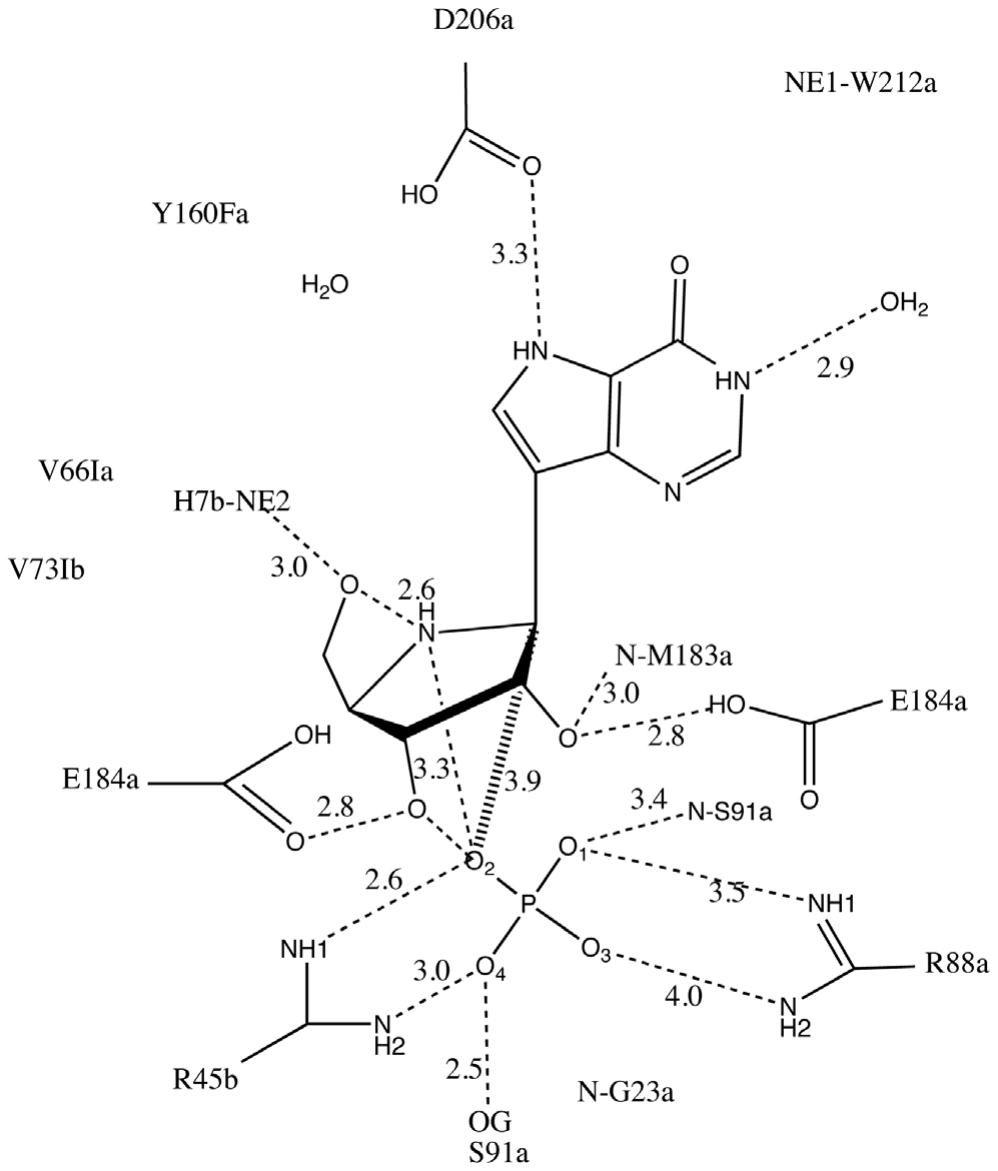
Catalytic site contacts for ImmH and PO_4_^3−^ at the active site of V66I:V73I:Y160F PfPNP. The schematic shows the catalytic site of the triple mutant Val66Ile:Val73Ile:Tyr160Phe PfPNP with ImmH and phosphate in the active site, which is at the interface of 2 subunits within the hexameric structure (a trimer of dimers). Amino acids are from the parent subunit unless labeled with *b*, which marks residues from the adjacent subunit. Dashed lines indicate hydrogen bonding. Distances are shown in Angstroms.

**Figure 7:**
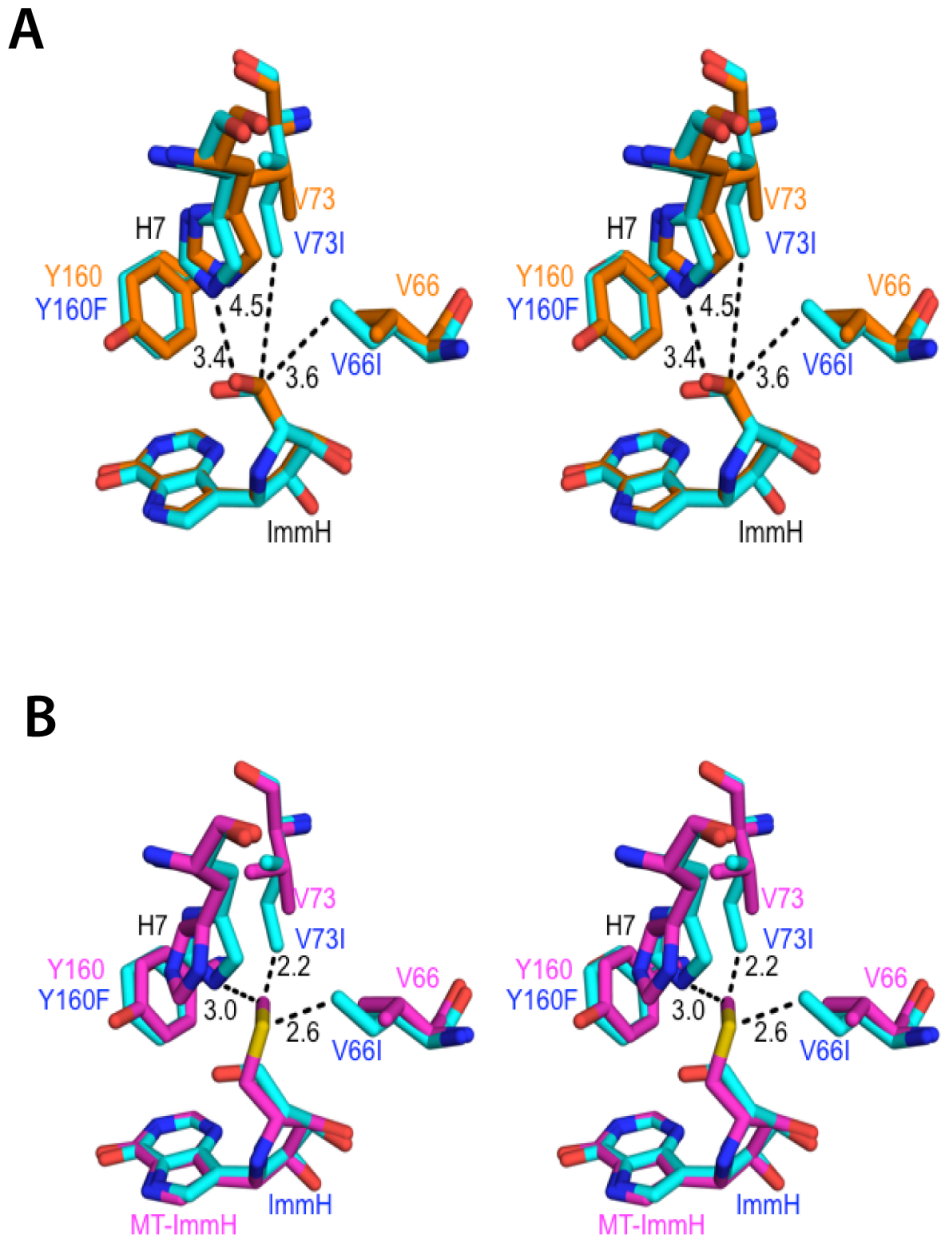
Cross-eyed stereo views of the superposition of Immucillins bound in the catalytic site of V66I:V73I:Y160F PfPNP. **A**) V66I:V73I:Y160F PfPNP with ImmH (PDB ID: 3FOW) (*blue*) overlays Wild type PfPNP with ImmH (PDB ID: 1NW4) in *orange*. **B**) An isolated overlay of V66I:V73I:Y160F PfPNP: ImmH *(orange)* and Wildtype PfPNP: MT-ImmH (1Q1G) (*purple*). The panel shows side chains in surrounding complex with MT-ImmH in the active pocket shifted relative to ImmH.

Cross-eyed stereo views showing residues that surround the transition state analogue inhibitor, ImmH, in V66I:V73I:Y160F PfPNP The figure was created using MacPyMol [23]. The parental monomer surrounding the bound ImmH (*green*), while the *yellow* side chains indicate residues contributed from the adjacent subunit (Figure 5a). There is a reduction of hydrogen bonds in V66I:V73I:Y160F PfPNP to wildtype PfPNP (Figure 5b). Tyr160, Val181, Met183, Asp206, and Trp212 surround the 9-deazapurine base of ImmH. The Tyr160Phe mutant is unable to participate in hydrogen bonding with water and Asp206 due to the lack of the hydroxyl group on the Phe side chain (Figures 6 & 7). Phosphate ion sits 4.5 Å under the O3′ hydroxyl group of ImmH in the catalytic pocket. Each phosphate oxygen participates in 2 hydrogen bonds with Ser91, Arg88, Gly23, and Arg45 from the adjoining subunit. Phosphate oxygen is 3.4 Å from the Ser91 side chain of V66I:V73I:Y160F PfPNP. In wild type PfPNP the sulfate oxygen interacts with ImmH O3′ [17], but in V66I:V73I:Y160F PfPNP, phosphate does not seem to interact with the 3′-hydroxyl of ImmH. The V66I:V73I:Y160F PfPNP pocket in the catalytic site near the 5′-hydroxyl group of ImmH is in closer proximity to CD1 of Val66Ile (4.7 Å), CD1 Val73Ile (5 Å), and CZ of Tyr160Phe (4.2 Å), than with wild-type PfPNP. Wild-type values as stated in Shi, *et al*, shows the distance of the ImmH 5′-hydroxyl group CD1 of Val66 (5.1 Å), CD1 Val73 (6.2 Å), and CZ of Tyr160 (4.6 Å) [17]. A water molecule is 3.8 Å from the 5′-hydroxyl group of ImmH in the PfPNP mutant active pocket. The O3′ and O2′ of the ImmH iminoribitol ring directly interact with a hydroxyl group located on the side chain of Glu184 (2.8 Å). His7 NE2 is 3 Å from the 5′-hydroxyl group of ImmH (Figure 6).

### Molecular Modeling of V66I:V73I:Y160F PfPNP with bound inhibitors

The V66I:V73I:Y160F PfPNP complex with MT-ImmH ligand was modeled based on the crystal structures of PfPNP with MT-ImmH using the Modeller 8v2 comparative modeling program. The model allowed for prediction of the accommodation of MT-ImmH in V66I:V73I:Y160F PfPNP catalytic pocket (Figure 7). The overlap of the ImmH and MT-ImmH shows the difference in the 5′ group jutting into the active site with representative residues. According to the comparative model with MT-ImmH, Val66Ile (CD1) is calculated to be 3.4 Å from the 5′methylthio group, whereas Val66Ile (CD1) is 4.7 Å away from 5′ hydroxyl group of ImmH (Table 4). Val73Ile (CD1) is calculated to be about 4.7 Å from the 5′methylthio group, compared to Val73Ile (CD1), which is 5 Å away from 5′ hydroxyl group of ImmH. The proximity of Tyr160Phe (CE2) is 4.1 Å to the MT-ImmH 5′ group, compared to (CE2) 5.2 Å for 5′ hydroxyl of ImmH. This model predicts that the 5′ group of MT-ImmH is more crowded in the active site relative to 5′ group of ImmH.

**Table 4:** V66I:V73I:Y160F PfPNP Mutant Residues R-Group Atom Distances to Immucillins.

Site directed mutant PfPNP	R-group atoms	ImmH PDB: 3FOW	MT-ImmH 5′-Methylthio Modeller 8v2 Simulated	Wild type PfPNP	R-group atoms	ImmH PDB:1NW4	MT-ImmH PDB:1Q1G
Val66lle	CD1	C5′ - 3.8 Å	CS′ - 3.4 Å				
	CG1	C5′ - 4.2 Å	CS′ - 3.9 Å	Val 66	CG1	C5′ - 4.1 Å	CS′ - 3.5 Å
	CG2	C5′ - 4.3 Å	CS′ - 5.8 Å		CG2	C5′ - 4.3 Å	CS′ - 4.7 Å
Val73Ile	CD1	C5′ - 4.4 Å	CS′ - 4.7 Å	Val 73	CG1	C5′ - 5.9 Å	CS′ - 3.8 Å
	CG2	C5′ - 6.4 Å	CS′ - 4.5 Å		CG2	C5′ - 5.9 Å	CS′ - 3.5 Å
Tyr160Phe	CZ	OH′ - 4.0 Å	CS′ - 4.0 Å	Tyr160	CZ	O5′ - 4.7 Å	CS′ - 4.5 Å
	CE1	OH′ - 3.4 Å	CS′ - 3.7 Å		CE1	O5′ - 4.2 Å	CS′ - 4.0 Å
	CE2	OH′ - 5.2 Å	CS′ - 4.1 Å		CE2	O5′ - 5.6 Å	CS′ - 4.1 Å
	CD1	OH′ - 4.0 Å	CS′ - 3.6 Å		CD1	O5′ - 4.8 Å	CS′ - 3.5 Å
	CD2	OH′ - 5.6 Å	CS′ - 4.0 Å		CD2	O5′ - 6.1 Å	CS′ - 3.6 Å
	CG	OH′ - 5.1 Å	CS′ - 3.7 Å		CG	O5′ - 5.7 Å	CS′ - 3.5 Å

R-group carbons of comparatively designed V66I:V73I:Y160F PfPNP:MT-ImmH and V66I:V73I:Y160F PfPNP:ImmH crystal structure distances from 5′ group of ImmH or MT-ImmH were measured in MacPymol. V66I:V73I:Y160F PfPNP:MT-ImmH based on the X-ray crystallography structure of *P. falciparum* purine nucleoside phosphorylase bound to MT-ImmH (Protein Data Bank ID: 1Q1G) [17]. V66I:V73I:Y160F PfPNP:MT-ImmH structure was generated utilizing MODELLER 8v2 program [21], [22].

## Discussion

Malaria parasite survival depends upon access to hypoxanthine obtained from direct uptake from the host environment or through the action of the *Plasmodium* purine salvage pathway. Since erythrocytes do not synthesize the polyamines that are essential for *Plasmodium*, malaria parasites are dependent on their own polyamine pathway for metabolic needs [31]. *Plasmodium* species need to metabolize MTA, the product of polyamine synthesis. MTA accumulation leads to feedback inhibition of polyamine synthesis, and has been shown to lead to antiproliferative effects [6], [32], [33]. MTA also represents an additional source of purines for *Plasmodium*.

PfPNP compensates for the streamlining of its non-redundant purine pathway by having multi-substrate activities. *Plasmodium* purine salvage enzymes, ADA and PNP, are unique in their ability to take either purines or 5′-methylthio purines. MTI is exclusively a *Plasmodium* metabolite that is neither produced nor metabolized in other Apicomplexa including *Toxoplasma* [18].

We investigated the residues in the catalytic pocket of PfPNP that interact with the 5′-methylthio group of MTI and transition state analogue MT-ImmH. We also created substrate binding site mutants of PfPNP with altered activity for inosine and MTI. V66I:V73I:Y160F PfPNP, a *P. falciparum* PNP mutant designed to mimic TgPNP, retains wild-type PfPNP efficiency with the inosine substrate, but has significantly reduced phosphorylysis of MTI. Residues that line the active site, such as Arg45 and Tyr47, are critical for enzyme function. Arg45 is conserved in family I PNPs including *Plasmodium*, *Trichomonas vaginalis*, *T. gondii*, and *E. coli* PNPs [17], [18], [29], [34], whereas Tyr47 is conserved in *Plasmodium* species and *T. gondii*. Asp206 has been implicated in hydrogen bonding with inosine and essential for purine base binding [2], [35].

*Plasmodium yoelli* PNP has Ile at position 75 that corresponds to PfPNP Val73, yet PyPNP has similar activity for both inosine and methylthioinosine as *P. falciparum* PNP [14]. As confirmed in mutagenesis studies, the Val73Ile substitution is not critical in reducing PfPNP activity for MTI, although it has a synergistic effect with the other mutations. Surprisingly, the Val73Ile PfPNP mutant binds less tightly to ImmH. The decreased sensitivity of the Val73Ile to ImmH and MT-ImmH is unexpected, since PyPNP is catalytically indistinguishable from PfPNP with similar *K_d_* for ImmH and MT-ImmH as PfPNP ([14] and unpublished).

For many of the PfPNP mutations that affect PfPNP catalytic activity, only a modest change in *K_m_* was observed (Tables 1 and 2). *K_m_* is the concentration of substrate at which half of the enzyme catalytic sites are filled by substrate in the steady-state. Binding of transition state inhibitors will reflect the energy of binding to the transition state. Assuming the chemical step is rate limiting, the effect of mutations that alter transition state binding would be expected to correlate with *k_cat_*. Thus, the data suggest that Tyr160 of PfPNP is particularly important for binding to the transition state. Comparison of *k_cat_/K_m_* illustrates the changes in catalytic efficiency of the mutant enzymes compared to the wild-type for MTI and inosine.

The crystal structure of V66I:V73I:Y160F PfPNP•ImmH•PO_4_^3−^ supports the results from the enzymology studies of the mutants. The crystal structures of wild-type PfPNP bound to ImmH and MT-ImmH differ in the position of the 5′-group of the inhibitor [17]. The 5′-methylthio group of MT-ImmH is rotated approximately 135° when compared to the 5′ hydroxyl of ImmH, allowing the 5′methylthio group to occupy a hydrophobic pocket in the active site that is different from the hydrophilic pocket that faces the 5′-hydroxyl group [17]. When ImmH is bound to the wild-type PfPNP active site, Tyr160, through a water-mediated contact, interacts with the 5′-hydroxyl group of the inhibitor. The Tyr160Phe in V66I:V73I:Y160F PfPNP cannot participate in the water-mediated interaction with the 5′-hydroxyl of ImmH due to the hydrophobic properties of the Phe160 side chain. Tyr160Phe mutation in PfPNP reduces the water-mediated hydrogen bonds with Asp206 and water molecules in the pocket. The Tyr160Phe mutation coupled with either Val66Ile or Val73Ile significantly reduces the efficiency of PfPNP for MTI.

Although the V66I:V73I:Y160F PfPNP•ImmH•PO_4_^3−^ structure is limited by its 2.8 Å resolution, there appear to be fewer water molecules found in the hydrophobic region of the catalytic site that coincides with the increased number of hydrophobic residues in the V66I:V73I:Y160F PfPNP hydrophobic pocket of the active site. The V66I:V73I:Y160F PfPNP triple mutations reduce the docking of the 5′-methylthio side pocket in the catalytic domain. In the wild-type PfPNP (1NW4) structure, Val66 (5.1 Å CG1 and 5.4 Å CG2), and Val73 (6.4 Å CG1, 6.6 Å CG2) methyl groups sit further away from the 5′-hydroxyl group of the iminoribitol region of ImmH when compared to the structure of V66I:V73I:Y160F PfPNP with ImmH (3FOW) (Val66Ile - 4.7 Å CD1 and Val73Ile - 5.0 Å CD1).

This work shows that it is possible to selectively modify MTI activity while retaining inosine activity of PfPNP. Tyr160 is a conservative substitution for the Phe residue present in TgPNP, *E. coli* PNP and human PNP. While Val66, Val73 and Tyr160 residues all contribute to the catalytic efficiency of 5′-methylthio activity of PfPNP, the Tyr160 is critical, and a Tyr160Phe substitution in PfPNP significantly diminishes PfPNP sensitivity to MT-ImmH. Similarly, the Val73Ser mutation has a dramatic effect upon MTI activity without loss of inosine activity, presumably because the hydrophobic pocket that accommodates the MT sidechain is disrupted by the hydrophilic OH of Serine.

New antimalarials are needed to subdue the increase of drug resistant parasites, which is part of a concerted global effort to control of the spread of the disease [36]. Ideally, new chemotherapeutic agents will specifically inhibit parasite targets without significantly affecting host metabolic pathways. Since there is a lack of redundancy in the *Plasmodium* purine pathway, and purine salvage is essential, a variety of compounds that target the purine salvage pathway are being investigated as potential antimalarials [37]. Our studies elucidate the molecular basis of differences in substrate affinities and differential susceptibility to immucillins between PfPNP and TgPNP. Since *Plasmodium* parasites develop resistance to antimalarials readily, our studies suggest that resistance could develop to antimalarials based on targeting the unique 5′-methylthio activity of PNP without compromising activity against inosine. These studies should aid in the future rational design of PfPNP inhibitors as potential antimalarials.

## Supporting Information

Figure S1 Secondary structure of wild-type and mutant PNPs. Circular dichroism of wild-type and mutant PfPNPs to compare secondary structure. Enzyme concentration for each run was 0.1 mg/ml (3.3 nM). Spectra was converted to molar ellipticity (θ) after subtracting the solution of 10% HEPES buffer in water for baseline measured at 4°C. Molar ellipticity conversion  =  millidegrees/(pathlength×number of residues×molar protein concentration). The spectra are represented as follow: WT PfPNP (*red* circle), V66I:V73I:Y160F PfPNP (*orange* square), Asp206Ala PfPNP (*yellow* diamond), Met183Ala PfPNP (*green* x), and Arg45Ala (*blue* cross).
(TIF)

Figure S2 Omit Fo-Fc map for V66I:V73I:Y160F PfPNP mutant with ImmH. Omit Fo-Fc map (red) contoured at 3σ. The resolution for this map is 2.8 Å. Figures were prepared with MacPyMol [23].
(TIF)

Table S1 Primers used for site-directed mutagenesis construction of PfPNP.
(DOCX)

Table S2 Data processing and refinement statistics for V66I:V73I:Y160F PfPNP crystal structure.
(DOCX)
