# Supplementary figures and images for: Structural Determinants of the 5′-Methylthioinosine Specificity of Plasmodium Purine Nucleoside Phosphorylase
Source: PLoS One. 2014 Jan 8;9(1):e84384. doi: 10.1371/journal.pone.0084384 (PMC3885546; doi:10.1371/journal.pone.0084384)

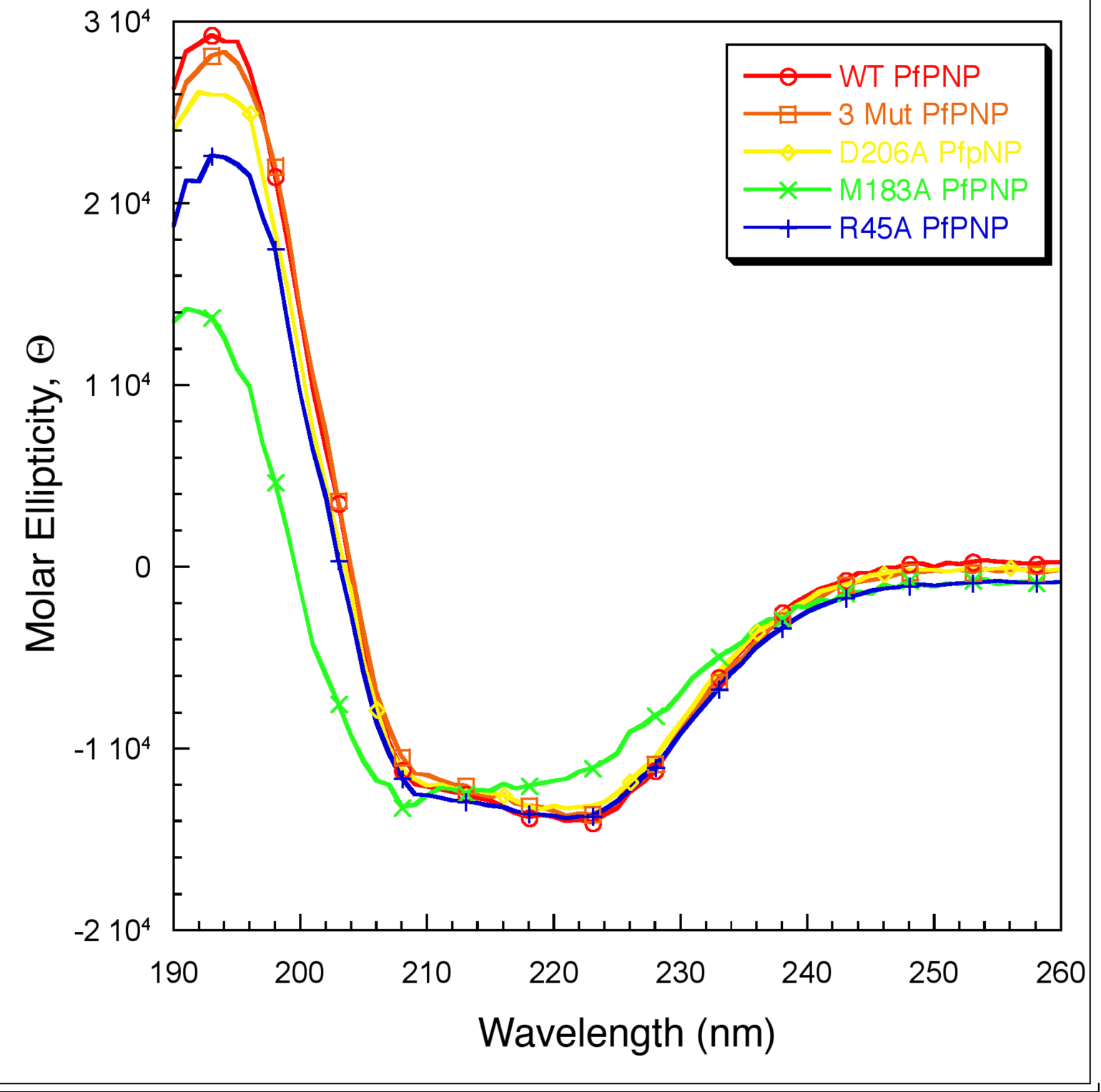

Supplement: Figure S1 — Secondary structure of wild-type and mutant PNPs. Circular dichroism of wild-type and mutant PfPNPs to compare secondary structure. Enzyme concentration for each run was 0.1 mg/ml (3.3 nM). Spectra was converted to molar ellipticity (θ) after subtracting the solution of 10% HEPES buffer in water for baseline measured at 4°C. Molar ellipticity conversion = millidegrees/(pathlength×number of residues×molar protein concentration). The spectra are represented as follow: WT PfPNP (red circle), V66I:V73I:Y160F PfPNP (orange square), Asp206Ala PfPNP (yellow diamond), Met183Ala PfPNP (green x), and Arg45Ala (blue cross). (TIF) [file pone.0084384.s001.tif]

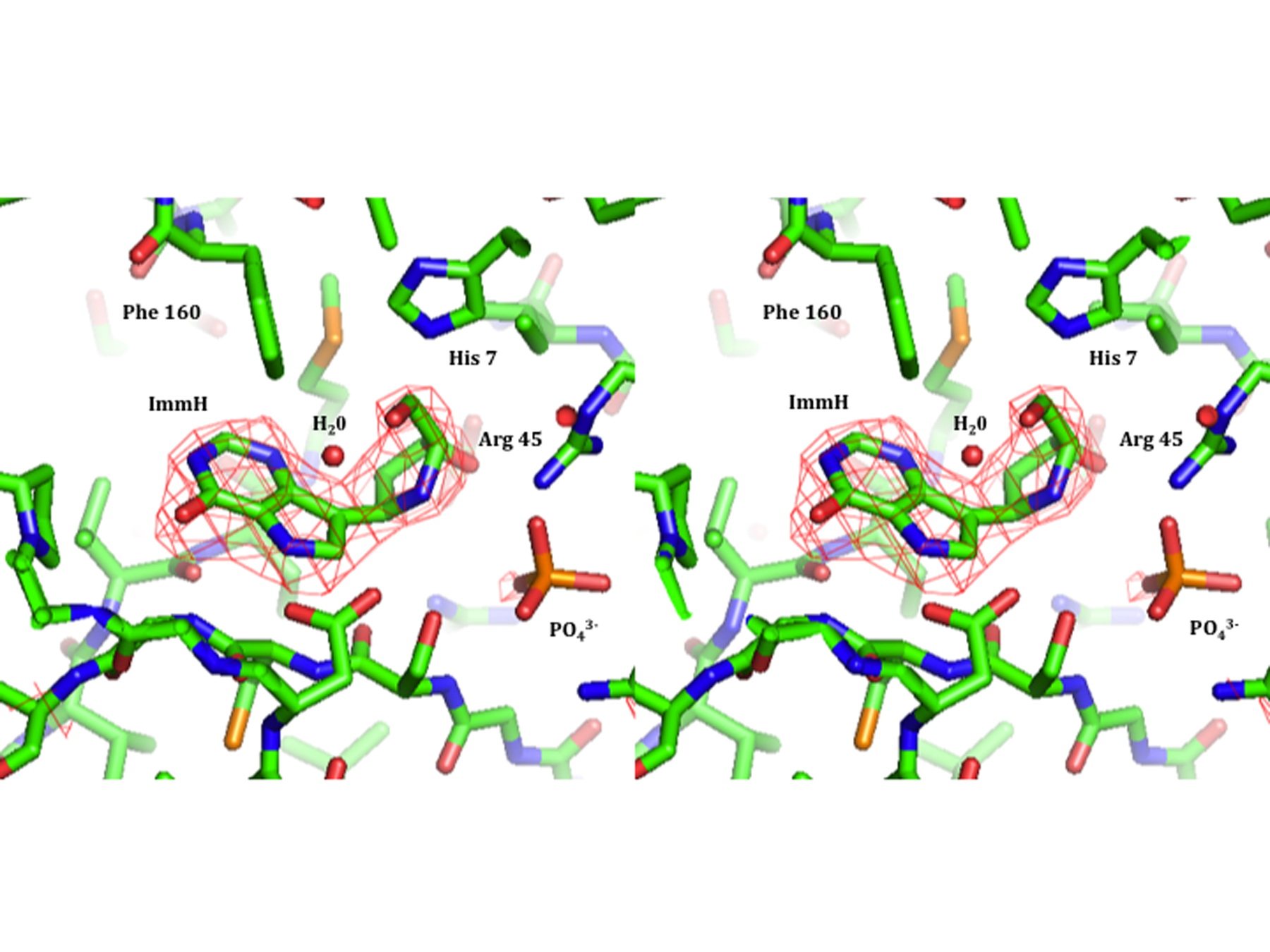

Supplement: Figure S2 — Omit Fo-Fc map for V66I:V73I:Y160F PfPNP mutant with ImmH. Omit Fo-Fc map (red) contoured at 3σ. The resolution for this map is 2.8 Å. Figures were prepared with MacPyMol [23]. (TIF) [file pone.0084384.s002.tif]
