# Supplementary material for: Structural Determinants of the 5′-Methylthioinosine Specificity of Plasmodium Purine Nucleoside Phosphorylase
Source: PLoS One. 2014 Jan 8;9(1):e84384. doi: 10.1371/journal.pone.0084384 (PMC3885546; doi:10.1371/journal.pone.0084384)
Supplement: Table S1 — Primers used for site-directed mutagenesis construction of PfPNP. (DOCX) [file pone.0084384.s003.docx]

**Supplemental Table 1.** Primers used for site-directed mutagenesis construction of PfPNP mutants.

| **Mutation** | **Primer** |
| --- | --- |
| His7Ala | 5′Phos-GCCCTTGATAATCTTTTACGC**GCT**TTAAAAATAAGCAAGGAAC-3’ |
| Tyr160Ala | 5′Phos-GGTATCAGTGTTTCATCAGATATG**GCT**TATCCCAATAAAATTATTCC-3’ |
| Asp206Ala | 5′Phos-GGTGGTATTCTTATTGTT**GCT**GGATGTCCATTCAAATGGGACG-3 |
| Arg45Ala | 5′Phos-CATATGTTGATTTAGCATACAAC**GCA**GAATACAAAAGTGTAGAATG-3 |
| Tyr47Ala | 5′Phos-GATTTAGCATACAACAGAGAA**GCC**AAAAGTGTAGAATGTC-3’ |
| Val73Ala | 5′Phos-GGTTCAGCAGGATGTGCT**GCA**TGTTTTGAAGAATTATGTC-3’ |
| Val66Ala | 5′Phos-GAAATTTTTATGTGTTAGTCACGGT**GCA**GGTTCAG-3’ |
| Met183Ala | 5′Phos-CTAAAGCTAATGCTGCTGTTGTTGAA**GCG**GAACTAGCCACTC-3’ |
| Val66Ile | 5′Phos-GAAATTTTTATGTGTTAGTCACGGT**ATA**GGTTCAG-3’ |
| Val73Ile | 5′Phos-GGTTCAGCAGGATGTGCT**ATA**TGTTTTGAAGAATTATGTC-3’ |
| Val73Ser | 5′Phos-GGTTCAGCAGGATGTGCT**TCA**TGTTTTGAAGAATTATGTC-3’ |
| Val73Phe | 5′Phos-GGTTCAGCAGGATGTGCT**TTT**TGTTTTGAAGAATTATGTC-3’ |
| His7Ser | 5′Phos-GATAATCTTTTACGC**TCT**TTAAAAATAAGCAAGGAAC-3’ |
| His7Phe | 5′Phos-GATAATCTTTTACGC**TTT**TTAAAAATAAGCAAGGAAC-3’ |
| Val66Ser | 5′Phos-GAAATTTTTATGTGTTAGTCACGGT**TCA**GGTTCAG-3’ |
| Val66Phe | 5′Phos-GAAATTTTTATGTGTTAGTCACGGT**TTT**GGTTCAG-3’ |
| Tyr160Phe | 5′Phos-GGTATCAGTGTTTCATCAGATATG**TTT**TATCCC-3’ |
| Tyr160Trp | 5′Phos-GGTATCAGTGTTTCATCAGATATG**TGG**TATCCC-3’ |
