# Supplementary material for: Structural Determinants of the 5′-Methylthioinosine Specificity of Plasmodium Purine Nucleoside Phosphorylase
Source: PLoS One. 2014 Jan 8;9(1):e84384. doi: 10.1371/journal.pone.0084384 (PMC3885546; doi:10.1371/journal.pone.0084384)
Supplement: Table S2 — Data processing and refinement statistics for V66I:V73I:Y160F PfPNP crystal structure. (DOCX) [file pone.0084384.s004.docx]

| Supplemental Table 2: Data Processing and Refinement Statistics for V66I:V73I:Y160F PfPNP | |
| --- | --- |
|  | V66I:V73I:Y160F PfPNP-ImmH |
| Resolution (Å) ^a^ | 50-2.8 (2.88-2.80) |
| Unit cell parameters (Å) | a=b=c=234.97  α=β=γ=90° |
| Unique Reflections | 27430 |
| Completeness (%)^a^ | 99.9 (99.9) |
| Redundancy ^a^ | 13.3 (7.7) |
| R _sym_ (%)^a^ | 11.7 (47.1) |
| *I/σ*^a^ | 22.9 (3.3) |
| # of protein atoms^b^ | 3559 |
| # of water molecules^b^ | 77 |
| *R*-factor^c^ (%) | 16.5 (25.5) |
| *R*_free_ (%) | 20.2 (32.8) |
| Average *B*-factor (Å^2^) | 44.2 |
| Ligand *B*-factor (Å^2^) | 43.04 |
| r.m.s.^d^ bond (Å) | 0.01 |
| r.m.s. angle (Å) | 1.50 |
| Ramachandran Analysis | |
| most favored | 86.9% |
| additional allowed | 12.6% |
| Generous allowed | 0.5% |
| Disallowed | 0% |
| ^a^ The values for the highest resolution shell are in parentheses.  ^b^ For the asymmetric unit.  ^c^ R factor = ∑_hkl_⎜⎜F_obs_⎜ - k⎜F_calc_⎜⎜/∑_hkl_⎜F_obs_⎜, where F_obs_ and F_calc_ are observed and calculated structure factors, respectively.  ^d^ Root mean square | |
